# Machine Learning in Biomarker-Driven Precision Oncology: Automated Immunohistochemistry Scoring and Emerging Directions in Genitourinary Cancers

**DOI:** 10.3390/curroncol33010031

**Published:** 2026-01-06

**Authors:** Matthew Yap, Ioana-Maria Mihai, Gang Wang

**Affiliations:** 1 Department of Pathology and Laboratory Medicine, University of British Columbia, Vancouver, BC V6T 1Z7, Canada; 2 Pathology and Laboratory Medicine, BC Cancer, Vancouver, BC V5Z 4E6, Canada

**Keywords:** precision oncology, machine learning, digital pathology, artificial intelligence, immunohistochemistry, automation, prognostic, predictive, biomarker

## Abstract

Immunohistochemistry (IHC) is essential for diagnostic, prognostic, and predictive biomarker assessment in oncology, but manual interpretation is limited by subjectivity and inter-observer variability. Machine learning (ML), a computational subset of AI that allows algorithms to recognise patterns and learn from annotated datasets to make predictions or decisions, has led to advancements in digital pathology by supporting automated quantification of biomarker expression on whole-slide images (WSIs). This review evaluates the role of ML-assisted IHC scoring in the transition from validated biomarkers to the discovery of emerging prognostic and predictive IHC biomarkers for genitourinary (GU) tumours. Current applications include ML-based scoring of routinely used biomarkers such as ER/PR, HER2, mismatch repair (MMR) proteins, PD-L1, and Ki-67, demonstrating improved consistency and scalability. Emerging studies in GU cancers show that algorithms can quantify markers including androgen receptor (AR), PTEN, cytokeratins, Uroplakin II, Nectin-4 and immune checkpoint proteins, with early evidence indicating associations between ML-derived metrics and clinical outcomes. Important limitations remain, including limited availability of training datasets, variability in staining protocols, and regulatory challenges. Overall, ML-assisted IHC scoring is a reproducible and evolving approach that may support biomarker discovery and enhance precision GU oncology.

## Simple Summary

Immunohistochemistry (IHC) is a common test used by pathologists to detect cancer biomarkers, which can help with diagnosis, prognosis, and treatment selection. However, IHC results can vary between laboratories and between observers. New digital pathology tools and artificial intelligence (AI), particularly machine learning (ML) techniques, can analyse stained tissue more consistently. This review gives an overview of how ML is being used to automate IHC scoring, first in well-studied biomarkers and then emerging biomarkers. This review then explores how these innovations can apply to genitourinary (GU) oncology, including prostate, renal, and bladder tumours, for which researchers have begun applying ML to new biomarkers that may predict outcomes or treatment response. ML use in IHC scoring is promising but requires more validation.

## 1. Introduction

Precision oncology depends on accurate biomarker detection and quantification to predict treatment response or determine patient eligibility for targeted therapies [1,2,3]. Immunohistochemistry (IHC) is traditionally assessed by visual scoring, which faces limitations in precision due to subjective threshold interpretation, inter-observer variability, and poor scalability [4,5,6,7]. Such constraints have inspired innovations in digital pathology and artificial intelligence (AI) to streamline clinical biomarker quantification [5,8].

Though liquid biopsy assays such as those detecting circulating tumour DNA are gaining traction in real-time, prognostic detection of disease burden and recurrence [9,10,11,12,13,14], their predictive role in guiding treatment decisions is limited and largely complementary to tissue-based testing [10,13,14]. IHC also provides spatial context and insights into tissue architecture and tumour–immune microenvironment interactions that correlate with certain tumour behaviours and treatment responses [15]. Moreover, as an accessible, relatively cost-effective method [6,16,17], IHC remains a clinical benchmark for diagnostic biomarker assessment [18,19,20,21,22], and is increasingly explored for prognostic and predictive applications [4,16,17,23].

Advances in whole-slide imaging, combined with machine learning (ML), now allow automated, reproducible IHC scoring, which is progressively adopted in molecular cancer research [24,25,26,27,28,29]. Deployment of automated scoring pipelines for some biomarkers has demonstrated strong concordance with expert pathologist scoring, achieving reliable clinical integration in breast [30,31,32,33,34,35,36] and lung [17,37,38,39,40,41,42] cancers. Beyond breast and lung oncology, AI-enabled IHC quantification is expanding, exploring potential applications in other common malignancies [5,43,44], notably GU cancers [9,45,46], whose heterogeneity has made prognostic and predictive biomarker panels difficult to standardise [9,45,47,48,49].

Building on recent advancements in computational pathology, this narrative review explores how automated IHC scoring can be translated into clinical routine. We summarise current applications and the state of evidence for ML use on emerging IHC biomarkers, with emphasis on GU oncology.

While numerous reviews have addressed AI in digital pathology, most have prioritised H&E-based histopathology, algorithmic methodology, or technical performance metrics. This review specifically examines ML-assisted IHC as a quantitative biomarker platform that positions GU malignancies as a logical next translational frontier, building on validated IHC scoring paradigms from other tumour types to contextualise emerging applications for GU oncology. By anchoring emerging GU applications to mature, well-studied ML–IHC frameworks, this review aims to explore the translational readiness, limitations, and future potential of automated IHC scoring in biomarker-driven GU precision oncology.

## 2. Methods

This narrative, non-systematic review was conducted in two stages, aiming to inductively identify themes from influential, emerging literature [Supplementary Figure S1]. The search was dynamic and iterative, using forward citation tracing to track evolving research trajectories, and was not intended to exhaustively extract or quantitatively pool data from a static snapshot of published works.

### 2.1. Stage 1: Exploratory Literature-Mapping to Identify Foundational ML–IHC Themes

An exploratory search was conducted primarily in PubMed, with support from Google Scholar to identify foundational literature between 2000 and 2025 (e.g., landmark articles, reviews) on digital pathology, ML, IHC, and biomarker quantification in precision oncology. Search terms included combinations of “artificial intelligence” or “AI”, “machine learning”, “immunohistochemistry” or “IHC”, “digital pathology”, “biomarker”, quantif* (quantification), cancer, tumor/tumour, malignanc*, neoplas*, “treatment response”, “therapeutic response”, “computational pathology”, “quantitative pathology”, “whole slide imaging” or “WSI”, “digital image analysis” and “automated”.

The scope of the literature search for this narrative review was limited to articles in which IHC was the primary analytical method and for which ML-based quantification was applied directly for biomarker interpretation, prognostic assessment or therapeutic decision-making, beyond technical metrics or solely image-processing objectives.

Within these bounds, 40 search results were screened by title and abstract. Articles were included if they applied AI or ML to IHC image analysis or automated biomarker quantification. Reviews relevant to digital pathology workflows were also included. Studies focused solely on non-IHC modalities, such as H&E, immunofluorescence, liquid biopsy, radiology, and transcriptomics, were excluded. Research unrelated to human solid tumours, as well as publications from non-oncology disciplines (e.g., veterinary medicine, dentistry, nutrition), was also excluded.

After full-text review, 12 core articles were retained, and reference snowballing expanded the pool to 64 papers, of which 20 involved ML-based IHC quantification. From this literature-mapping, five biomarkers (ER/PR, HER2, MMR, PD-L1, and Ki-67) emerged inductively as the most recurrently studied ML–IHC targets [Supplementary Table S1]. These biomarkers are synthesised in Section 4 as validated biomarker exemplars of automated IHC scoring. Additional studies were then identified through reference chaining and forward “cited by” tracing.

### 2.2. Stage 2: Targeted Narrative Exploration of ML Use for IHC Biomarkers in GU Cancers

Building on Stage 1 findings, a targeted narrative search was performed to examine the recent applications of ML-enabled IHC scoring within GU cancers. To complement database querying, we additionally used citation tracing and forward “cited by” exploration from foundational papers to identify recent (2020–2025) representative studies extending ML–IHC frameworks to GU contexts.

Study inclusion criteria were restricted to articles investigating prostate, bladder, and renal malignancies that applied digital pathology or ML workflows for the quantification of IHC biomarkers associated with prognosis or therapeutic response. Articles were excluded from the synthesis if they relied solely on other modalities, such as H&E, radiomics, or transcriptomics, without an IHC component, or if they only used manual analysis.

Furthermore, we excluded studies focused exclusively on diagnostic classification, molecular subtyping, or technical metrics lacking translational relevance to clinical outcomes. Investigations detailing purely technical ML workflows, such as nuclear segmentation without biomarker scoring, and those utilising IHC merely as a confirmatory reference rather than the primary analytical target were also omitted. Stage 2 of the narrative search prioritised studies demonstrating translational relevance, emerging biomarker development, or method validation, rather than attempting to catalogue all GU-associated markers. The objective of this search stage was to highlight domains in which ML-assisted IHC is actively evolving within GU oncology, including early, pre-validation exploratory models.

## 3. IHC Scoring Variability and the Need for ML-Assisted Analysis

IHC is a technique that enables visualisation of protein expression within preserved tissue architecture, providing spatial information about biomarker distribution and tumour–microenvironment interactions [4,6,16]. In clinical and research settings, interpretation typically relies on semiquantitative scoring systems that assess localisation, staining intensity, and the proportion of positive cells [7,21,50,51]. Common IHC scoring approaches include ordinal scales, proportion-based metrics, and composite scoring systems such as Allred-type and Histologic Score (H-score) methods [7,21,50,51].

Even with established scoring systems, reproducibility remains a challenge. IHC scoring cut-offs can vary across laboratories due to differences in staining protocols, tissue processing, antibody clones, scanner calibration and pathologist interpretation [50,51,52,53]. Inter-observer discordance further increases with heterogeneity and borderline cases [50,51,52,53]. These limitations highlight the need for computational approaches that improve consistency and scalability in IHC interpretation [24,26,54] (Table 1).

WSI scanning technology has enabled the digitisation of entire slides at diagnostic resolution, supporting remote review, archiving, and digital image analysis (DIA) [24,25,65,66]. Early IHC quantification tools based on pixel thresholds and colour deconvolution provided approximate scoring but struggled to generalise across staining and batch variation [24,29]. With increasing digital adoption, ML methods are positioned to enhance reliability in IHC scoring [8,67]. Deep learning frameworks such as convolutional neural networks (CNNs) and multiple instance learning (MIL) can extract cellular and spatial features directly from WSIs, enabling automated detection of nuclei, tissue compartments, and chromogenic signal patterns [28,54].

In current human-in-the-loop workflows, ML algorithms function as assistive tools to pre-segment nuclei, quantify staining intensities, and flag regions of interest, prior to expert pathologist review and judgement [26,54,68]. These workflows reduce manual burden and variability for pathologists, while their expert feedback is used to further refine model performance [24,27]. ML-based scoring increasingly demonstrates agreement with expert assessment and provides a foundation for scalable, automated biomarker quantification [26,38,54,60].

Validated IHC biomarkers with established scoring frameworks and annotated datasets can thus serve as exemplars for the development of automated pipelines [26,51,52]. The next section highlights how these established biomarkers have been leveraged to demonstrate the feasibility and translational readiness of ML-based IHC quantification.

## 4. Validated Biomarkers as Models for ML-Based IHC Interpretation

Beyond diagnostics, some widely adopted biomarkers have established the prognostic and predictive utility of IHC in guiding precise treatment decisions (Table 2). These biomarkers also have well-annotated datasets and established scoring frameworks that allow ML models to be trained, benchmarked and validated [8,69,70,71,72] in ways that are still being explored for many emerging IHC biomarkers. Building from these established markers, ML aims to standardise IHC scoring and enhance the reproducibility of digital pathology for quantitative, precision oncology.

### 4.1. Hormone-Receptor IHC

Estrogen and progesterone receptors (ER/PRs) are nuclear IHC biomarkers that define hormone-receptor-positive breast cancers and determine eligibility for endocrine treatments such as tamoxifen, aromatase inhibitors, or fulvestrant [55,75]. Clinical evidence indicates that higher Allred scores can predict better outcomes and greater benefit from endocrine therapy [55,75]. For example, higher Allred scores in breast cancer patients have been associated with better five-year survival [55]. Traditional visual scoring of ER/PRs by pathologists is limited by interpretive variability, particularly in low-positive and heterogeneous cases, leading to inconsistencies in the quantification of nuclear staining [7,22,76]. ML-based scoring pipelines automating intensity thresholding and nuclear segmentation have shown close agreement with expert scoring in retrospective datasets, with improved inter-observer concordance and reduced turnaround time, despite differences in staining and scanning equipment [26,27]. Although ER/PR testing is specific to breast cancer [56], these validated frameworks provide a reference for standardising the semi-quantitative scoring of other nuclear biomarkers. For example, similar intensity-proportion scoring pipelines have been applied to nuclear biomarkers in bladder cancers for subtyping and prognostication [77,78,79,80]. These applications are revisited in Section 5.3.

### 4.2. Growth-Receptor IHC

ML-assisted quantification in ER/PR analysis has similarly extended to Human Epidermal Growth Factor Receptor 2 (HER2) IHC, a membranous biomarker whose scoring is used for determining eligibility for anti-HER2 therapy [57]. HER2 IHC assesses membranous expression using an ordinal scale (0, 1+, 2+, 3+) with confirmatory in situ hybridisation (ISH) for 2+ cases [56,57]. Distinguishing between HER2-negative (0) and HER2-low (1+) tumours has become increasingly important, as HER2-low cases comprise more than 50% of breast cancers and HER2-low status serves as a clinically validated, predictive biomarker of benefit from trastuzumab deruxtecan treatment [58,81]. Visually making this distinction, however, can remain challenging even for experts [57].

Now, ML models trained on curated HER2 datasets have achieved area-under-the-curve (AUC) values above 0.9 for differentiating HER2-low status from negative, highlighting the technical feasibility of reproducible assessment for weak or incomplete membranous stains [28,57]. Before routine adoption, however, such models must demonstrate robustness across antibody clones and laboratories [50,56]. The methodological principles of HER2 quantification are relevant to membranous and cytoplasmic biomarkers in GU oncology, with emerging prognostic and predictive value [77,80].

### 4.3. Mismatch-Repair (MMR) IHC

While HER2 and ER/PR IHC quantification is built on ordinal and continuous scoring schemes, ML-based approaches have likewise been applied to binary testing defined by loss or retention, such as MMR proteins [59,82,83]. MMR IHC is used to screen for microsatellite instability (MSI), a predictive marker of response to immune checkpoint inhibitors (ICIs) targeting immune cell Programmed Death receptor-1 (PD1), such as pembrolizumab and nivolumab [59,82,83,84]. Loss of nuclear staining for MLH1, PMS2, MSH2 or MSH6 indicates deficient mismatch repair (dMMR), whereas preserved expression of MMR defines proficiency (pMMR) [83]. MMR IHC is routinely performed on colorectal and endometrial carcinomas as part of Lynch syndrome screening and MSI assessment, with well-defined clinical thresholds [59,83,85]. Though its interpretation in clinical routine remains manual, CNN-based nuclear segmentation and classification models have shown concordance with expert identification of nuclear MMR loss vs. retention [73,86]. Validation studies have reported concordance rates above 90% in classifying tumours on WSIs as dMMR or pMMR [74,87].

ML automation of MMR loss detection highlights the potential of integrating ML-assisted workflows for high-throughput nuclear stain assessment. While MSI status is best understood as a key biomarker in colorectal and endometrial cancers, its potential role as a predictive biomarker for ICI response is now being explored within GU oncology workflows [88,89,90,91]. MMR IHC is an area of research interest in prostate cancers and upper tract urothelial carcinomas (UTUCs), for which dMMR or MSI-high status could inform hereditary cancer risk and predict response to ICI therapies [92,93,94,95]. MSI-high/dMMR status in UTUC in particular has been linked to Lynch-syndrome-related disease, supporting the use of MMR IHC in ICI selection, as well as flagging patients for germline testing [93,94,95]. Similarly, small retrospective studies have reported that dMMR prostate tumours, often with higher mutational burden, demonstrate durable response to PD-1 blockade (e.g., pembrolizumab) in select patients [92]. These applications of MSI-high/dMMR status require larger-scale validation in prostate tumours pending standardisation, with which AI-automated analysis may help, as revisited in Section 5.1.

### 4.4. Immune-Checkpoint IHC

While MMR IHC serves as a proxy for predicting checkpoint inhibitor response by genomic instability, IHC is also used directly on tumour-expressed Programmed Death Ligand-1 (PD-L1), which binds to the immune cell receptor PD-1 [39,59,84,96]. PD-L1 IHC is an established predictive biomarker of response to ICIs across tumour types, including non-small-cell lung cancers (NSCLCs) [64,97]. PD-L1 expression is assessed using one of three scoring systems: Tumour Proportion Score (TPS), Combined Positive Score (CPS) or Immune Cell (IC) score, with each system requiring specific spatial context and cell classification [97]. This complexity makes PD-L1 IHC challenging to quantify reproducibly by manual IHC interpretation around relevant cutoffs (e.g., CPS 1 or 10) [59,64]. ML approaches have accordingly been developed to automatically classify cell objects, segment tumour, immune and stromal compartments, detect 3,3′-Diaminobenzidine (DAB)-positive nuclei and membranes, and calculate TPS or CPS [39,64]. ML-based scoring of PD-L1 IHC has demonstrated improving agreement with expert consensus in validation studies, reporting intraclass correlation coefficients (ICCs) around the ~0.8 to 0.9 range, though agreement metrics vary by assay, tumour type, and scoring system. Inter-assay and inter-laboratory reproducibility remains a challenge for both manual and ML-based PD-L1 interpretation, though ML is likely to continue improving.

For example, a recent weakly supervised deep learning model using a 22C3 assay for PD-L1 tumour proportion scoring in NSCLC reported an ICC of 0.96 versus pathologist assessments in internal and external validation cohorts [63]. Beyond lung cancers, some immunotherapies have also been implemented in standard of care for renal and bladder cancers, in which immune infiltration and histologic heterogeneity add complexity to interpretation [64,98]. ML-based PD-L1 quantification thus highlights the potential of automated IHC scoring to extend beyond intensity thresholding toward context-aware, cell-type-specific biomarker analyses as ML models improve.

### 4.5. Proliferation IHC

Comparable ML-based approaches are underway for Ki-67 IHC, another established cancer biomarker [44]. Ki-67 is a nuclear marker of proliferation used for prognostication and grading in breast malignancies and neuroendocrine tumours (NETs) [8,27,52,99]. Higher Ki-67 indices correlate with more aggressive disease and worse outcomes [29,44,60,100,101,102]. In breast cancers, Ki-67 is used for the luminal A and luminal B phenotypes to guide adjuvant chemotherapy decisions [103,104]. In NETs, the Ki-67 index is part of the WHO G1–G3 grading system that directs therapy [61,105].

Manual counting of nuclear positivity on printed images is historically a common method of Ki-67 quantification, but this method can be labour-intensive and inconsistent. A reproducibility study found inter-laboratory intraclass correlations of 0.59 to 0.71 for Ki-67 visual scoring across eight laboratories [52]. In 2020, The International Ki-67 in Breast Cancer Working Group highlighted the difficulty of standardising Ki-67 IHC, noting that automated scoring could help overcome limitations [51]. Recent studies have accordingly attempted to develop and assess ML-based Ki-67 index calculation. A nuclear segmentation algorithm reported 93% accuracy in segmenting cell nuclei, with a 2.1% error margin for detecting Ki-67 positivity [62]. Another study showed that automated analysis provided greater specificity, concordance and prognostic correlation compared to manual scoring for Ki-67 in breast cancer, with 10% fewer misclassifications, 5.5% more concordance and a significantly higher prognostic value for overall survival (*p* = 0.044), as measured with the Likelihood Ratio Chi-square [27].

Automated pipelines built on deep learning nuclear segmentation architectures such as U-Net, Hover-Net and StarDist enable reliable nuclear identification and classification of cell positivity within region-of-interest selections, with stromal and necrotic exclusion [54,106]. ML models using deep learning nuclear segmentation can generate reproducible Ki-67 indices with strong correlation with pathologist counts (Pearson r > 0.9), while substantially reducing analysis time [8,102]. In GU malignancies, Ki-67 positivity has been associated with recurrence risk in prostate cancer and muscle invasiveness in bladder cancer [8,29,102], though clinical cut-offs remain less standardised [8,102]. Algorithmic quantification could facilitate reproducibility in multi-centre trials if integrated with harmonised protocols [8,44,85].

### 4.6. From Automated Scoring to Clinical Translation

Across ER/PR, HER2, MMR, PD-L1 and Ki-67, ML-based IHC quantification has achieved technical comparability with manual interpretation in some retrospective and multi-institutional validation studies. These validated IHC biomarkers illustrate the trajectory of ML in IHC: from reproducible IHC scoring towards integration of quantitative readouts into clinical decision-making. These translational exemplars provide a methodological and validatory foundation for extending automated scoring to additional biomarkers, including emerging prognostic and predictive GU biomarkers, as discussed in the following section.

## 5. Emerging Translational Applications of ML-Based IHC Scoring: An Evolving Frontier in GU Oncology

Applications of ML-assisted IHC quantification in GU oncology are a growing and evolving research focus [49]. GU tumours affecting the bladder, prostate, kidney, and related organs are highly heterogeneous, which has previously driven heavier research emphasis on diagnostic markers and molecular subtyping [49,80]. IHC biomarkers are central to routine diagnostic practice in GU malignancies, but robust prognostic and predictive panels have yet to be established [107,108]. Recent reviews have detailed the growing use of AI and digital pathology in GU malignancies, but many of these efforts have focused primarily on H&E-based histopathology and subtype classification [80,109].

Few studies have specifically examined the prognostic and predictive potential of ML-based IHC quantification across the broader spectrum of GU malignancies. Research has begun shifting from demonstrating quantitative accuracy to establishing meaningful thresholds for clinical utility. The emerging biomarkers discussed in this section describe how digital IHC is evolving from assisted quantification to prognostication and treatment response prediction, with implications in GU oncology (Table 3).

### 5.1. Prostate Cancer IHC

In prostate cancers, several biomarkers are under investigation for ML-based IHC interpretation. Automated scoring of androgen receptor (AR) expression is being explored for its potential association with lineage plasticity and treatment resistance. Phosphatase and tensin homolog (PTEN) loss assessed by binary IHC scoring, which has prognostic implications and correlates with phosphatidylinositol 3-kinase (PI3K) signalling pathway activation, is currently being evaluated using ML-based nuclear segmentation [53]. p63, an established basal cell marker used diagnostically, is also increasingly being examined in digital workflows for its potential subtype relevance [77]. Prostate-specific membrane antigen (PSMA), which is highly expressed in prostate tumours and underlies radioligand therapy, is being digitally quantified in exploratory ML-based workflows to assess expression patterns relevant to therapeutic targeting. B7 Homolog 3 (B7-H3), an immune checkpoint protein explored as a tumour-associated target in prostate cancer, is likewise being evaluated in early ML-enabled quantification approaches [113,117]. Early ML pipelines show improving performance in analyses with membranous and cytoplasmic staining patterns [121].

In addition, ML-based automation of MMR IHC binary classification represents a logical next step to explore and validate the prognostic and predictive applications of dMMR or MSI-high status in prostate cancers. Recent published AI studies on MSI status in prostate tumours have primarily focused on predicting MMR status from H&E slides, with manual IHC as the reference standard. But ML-driven workflows aimed at standardising the confirmatory binary IHC test itself have already been validated in colorectal cancers [74,86,87]. In principle, similar pipelines can be adapted to MMR IHC in prostate cancers. Specifically, automated single-cell nuclear detection of MLH1, PMS2, MSH2 and MSH6 loss has achieved near-perfect concordance with expert assessment (AUC ≈ 0.98) in colorectal tumours [73,87]. Adapting validated models trained on colorectal histology can steer AI research beyond H&E to standardising binary IHC for prostate pathology, enabling reproducible outputs for robust prognostic and predictive clinical correlations.

### 5.2. Renal Cancer IHC

Renal cancers have seen some progress in ML-based IHC quantification, with emerging prognostic applications [112,114]. Quantitative IHC of immune markers in Renal Cell Carcinoma (RCC) specimens is currently being integrated into exploratory prognostic models of immunotherapy response [114]. ML-based frameworks for WSI IHC analysis are being developed for the automated detection of BRCA1-Associated Protein 1 (BAP1) loss, which has retrospectively been associated with higher tumour grade, increased metastatic potential, and poorer clinical outcomes in clear cell RCCs [113]. V-domain immunoglobulin suppressor of T cell activation (VISTA), another immune checkpoint protein expressed in subsets of RCC and studied for immunotherapy resistance phenomena, is emerging as a potential, exploratory ML-quantifiable IHC biomarker [112,114,115]. These prognostic associations in RCC IHC, however, are based on surrogate endpoints and lack prospective clinical validation [80]. Building on frameworks developed for PD-L1, other immune checkpoint markers such as B7-H3, T-cell immunoglobulin and mucin-domain containing-3 (TIM-3) and Lymphocyte Activation Gene 3 (LAG-3), along with VISTA, are also under active research as potential predictive biomarkers for immunotherapy response in renal cancers, as well as bladder cancers [115,116,117,122,123].

### 5.3. Bladder Cancer IHC

Among GU malignancies, urothelial carcinoma of the bladder has received considerable attention in computational AI and IHC pathology research, which investigates IHC biomarkers with emerging prognostic and predictive utility [44,48].

Cytokeratin (CK) profiling is an important element of molecular subtyping in bladder cancers. CK5/6 and CK14, which are often co-expressed with the basal/squamous nuclear marker p63, define basal-like subtypes associated with aggressive behaviour and increased sensitivity to platinum-based chemotherapy [49,77,80]. CK20, similar to GATA3, is a marker of luminal differentiation, which is typically linked to more favourable prognosis and response to immunotherapy [49,109].

ML-based quantification of CK expression through automated nuclear and cytoplasmic segmentation is being used in subtyping [77]. Multi-institutional studies employing tissue microarrays constructed from retrospective cohort specimens have shown that ML-quantified CK5/6 and CK20 expression strongly correlates with transcriptomic classifications and clinical outcomes. Similar ML-based workflows have been applied to predict neoadjuvant chemotherapy (NAC) response in muscle-invasive bladder cancer (MIBC), by associating ML-quantified CK and immune markers with clinical variables [124].

Uroplakin II (UPK II), another bladder-specific marker, is a highly specific marker of urothelial differentiation with diagnostic use in determining tumour tissue-of-origin, and is being explored for its emerging prognostic and predictive value [9]. Digital quantification of UPK II staining intensity and distribution is currently aimed at distinguishing between primary bladder carcinomas and metastases while refining molecular subtype assignments, but is also under investigation for potential correlations with prognosis and treatment response in retrospective analyses [80].

Some therapeutically actionable surface markers are also currently being studied in bladder cancers. Trophoblast cell-surface antigen 2 (Trop-2), a transmembrane glycoprotein, and Nectin-4, a cellular adhesion molecule abundant in urothelial carcinomas, are being explored as candidate predictive markers to investigate associations with risk stratification and response to antibody-drug conjugates (ADCs), e.g., sacituzumab govitecan and enfortumab vedotin, respectively [118]. For Nectin-4 in particular, quantitative Nectin-4 IHC has shown retrospective associations with clinical outcomes, though predictive validation for treatment response remains to be established [9,118,119,120]. While high Nectin-4 expression is frequently assessed in the context of enfortumab vedotin treatment, thresholding practices vary and are not yet globally standardised [9,118]. Greater Nectin-4 expression, however, has been reported in aggressive urothelial carcinoma tissue variants, suggesting potential prognostic relevance [49,113,120].

Independent studies have found that greater Nectin-4 expression correlates with papillary morphology, lower tumour grade, and earlier pathological stage, with more favourable outcomes associated with predominantly membranous staining [120]. Recently, a QuPath-based ML workflow using a Random Trees classifier for tumour-cell classification and automated H-score computation was applied to quantify Nectin-4 intensity and assess spatial distribution and prognostic correlations in MIBC [119]. This study reported that tumour-front-enriched expression was associated with more favourable disease-free survival (DFS), while tumour-centre downregulation correlated with higher recurrence risk [119]. These findings support hypothesis generation but require validation in prospective, treatment-stratified cohorts. Although the QuPath workflow was not designed to validate Nectin-4 as a prognostic or predictive biomarker directly, it highlights how ML-assisted IHC quantification can offer reproducibility and standardisation to support digital pathology workflows requiring robust quantification.

This application of ML demonstrates that automated IHC scoring can support efforts to capture the spatial biology of tumours, which may have prognostic associations with clinical surrogate endpoints such as DFS [119]. Nectin-4 expression also aligns with luminal differentiation markers and shows inverse associations with PD-L1 expression on tumour-infiltrating immune cells, suggesting its integration within broader immune-microenvironment contexts [120]. Specifically, ML-assisted quantitative IHC pipelines are being explored in Cluster of Differentiation (CD) marker analysis to support immune profiling and to map immune infiltration patterns within bladder cancer tissue [44,49,64]. ML-enhanced quantitative IHC can improve the reliability of immune infiltration metrics, which are currently being investigated for potential associations with outcomes in MIBC patients receiving trimodal therapies aimed at bladder preservation [44,49,125].

### 5.4. Toward Clinical Translation and Therapeutic Action in GU Precision Oncology

As summarised in Table 3, most associations with clinical outcomes and evidence for ML applications in GU IHC remain retrospective and exploratory, with only few applications in select biomarkers approaching early clinical evaluation. Even for therapeutically actionable target markers, existing ML-based IHC workflows primarily support hypothesis generation rather than treatment selection.

The current state of evidence demonstrates a translational gap between technical feasibility and clinical implementation, highlighting the potential of ML-based methods to support future integration of reproducible GU IHC biomarker scoring into clinical decision-making. Notable biological heterogeneity across GU tumour sites, histologic variants and treatment contexts also further challenges model generalisability, underscoring the need for tumour-specific validation.

Current efforts at automating GU cancer IHC biomarker quantification support the feasibility of large-scale retrospective and prospective validation studies to improve discrimination between treatment responders and non-responders. As ML-based IHC quantification matures, emerging efforts in GU oncology are positioned to accelerate translation, following the roadmap established by frameworks in breast, lung, and gastrointestinal oncology. The emerging and growing emphasis on GU tumours suggests that ML-based IHC quantification can move beyond reproducing manual reads toward prognostication and treatment response monitoring. Realistic next steps toward clinical translation would involve multi-centre reproducibility studies and consensus on scoring thresholds, particularly for borderline or continuous biomarkers, to demonstrate robustness across laboratories, scanning systems and staining platforms.

## 6. Future Directions

ML-assisted IHC is moving beyond proof-of-concept studies toward translational adoption [110,126,127]. Future progress will depend on achieving reproducible IHC quantification methods for various experts across institutions that use varying staining platforms and scanners. Developing harmonised validation criteria and regulatory guidelines for software as a medical device will be essential to ensure reliability, transparency, and safe integration into clinical routine [71,111].

A key barrier to widespread implementation is the limited availability of large, diverse, and well-annotated IHC datasets across tumour types [72,110,127]. Broader data sharing and federated learning approaches, together with the creation of public benchmark datasets similar to CAMELYON in digital breast pathology, could accelerate evaluation and regulatory review [72,110,127]. This pathway of ML model validation for clinical use has been demonstrated in colorectal oncology as well. Specifically, the success of the Immunoscore showed that spatial immune metrics can achieve clinical use [128,129,130] with multi-centre and prognostic validation, suggesting that similar ML-driven spatial IHC analysis may be valuable to GU oncology [44,48,112].

On this path forward, research will likely prioritise interpretability and calibrated confidence outputs to support clinical trust and decision-making [43,110,126,127]. Progress is expected toward multimodal frameworks that integrate IHC-derived spatial features with genomic, transcriptomic, and clinical data [17,70,127]. For example, evidence from the IMvigor010 and IMvigor011 trials suggested that combined assessment of dynamic biomarkers such as ctDNA with histologic features may refine adjuvant therapy selection after cystectomy [131,132]. Under appropriate regulatory oversight, such composite approaches to integrating automation into IHC pathology could ultimately support more robust prognostic and predictive applications of AI for precision oncology.

## 7. Conclusions

This review demonstrates that although ML-based IHC scoring has gained traction in breast, lung, and gastrointestinal cancer research, its application in GU oncology remains largely exploratory. By outlining persistent barriers such as interobserver scoring variability, limited benchmark datasets, and the absence of harmonised cut-offs, we identify where ML-driven quantification could improve reproducibility, enable finer spatial and phenotypic analysis than manual methods, and support the optimisation or discovery of clinical IHC biomarkers. The novelty of this review lies in positioning GU malignancies as the next logical frontier for ML applications in IHC pathology, drawing on validated precedent frameworks to demonstrate how AI automation can enhance research efforts at targeted therapy, with continued development of multi-centre validation and reproducibility standards. As GU biomarker research accelerates, ML-driven IHC analysis offers a feasible pathway toward standardisation and scalability in tissue-based testing, with the potential to bridge current gaps between histopathology and molecular profiling in the digital era of precision oncology.

## Figures and Tables

**Table 1:** Clinical Validation Status and ML Compatibility of Common IHC Scoring Frameworks.

Scoring System	Context	Output	ML Relevance	Validation	Considerations	References
Allred	ER/PR	0–8	Well-suited for nuclear segmentation	Clinically validated and standardised	Well-established thresholds, but scoring can differ between observers	[31,55,56]
HER2 (ordinal) score	HER2	0/1+/2+/3+	Membrane modelling (CNN)	Clinically validated and standardised	Confirmatory ISH required for equivocal 2+ cases, but actionable HER2-low status	[27,30,31,34,57,58]
H-Score	Cytoplasmic/membranous IHC markers	0–300	Continuous intensity–proportion modelling	Exploratory; (research grade only)	No standardised thresholds for clinical use yet	[50,54,59]
Ki-67 Index	Proliferation (Breast, Colorectal, Neuroendocrine, Prostate, etc.)	% positive nuclei	Nuclear detection + % quantification suitable for ML automation	Clinically validated but with limitations in reproducibility	AI/ML can help standardise with variability in thresholding and counting	[51,52,60,61,62]
TPS	PD-L1	% tumour cells	Tumour-cell classification	Clinically validated, but thresholds depend on assay	Used in drug approval (tumour- & assay-dependent)	[37,39,40,41]
CPS	PD-L1	Combined tumour + immune	Multi-cell classification	Clinically validated but with limitations on reproducibility	Complex and variable; ML can help standardise	[39,42,63]
IC Score	PD-L1	% immune area	Region-based segmentation	Exploratory	Challenging for both manual and ML scoring	[41,42,64]

**Table 2:** Clinical Integration of ML-Driven Scoring Workflows for Validated IHC Biomarkers.

Validated Biomarker	Cancer Type	IHC Staining Pattern	Scoring System	ML Workflow	Clinical Role	ML Integration in Clinical Routine	References
ER/PR	Breast	Nuclear	Allred	Nuclear segmentation; intensity classification	Diagnostic; Predictive	validated; integrated	[31,55,56]
HER2	Breast; Gastric	Membranous	Ordinal	CNN; instance segmentation; membrane detection;	Diagnostic; Predictive (response to HER2-targeted + ADC therapy)	validated; integrated	[27,30,31,34,57,58]
MMR proteins	Colorectal; Endometrial; Prostate (emerging)	Nuclear	Binary (+/−)	CNN nuclear classification; MSI prediction	Diagnostic; Predictive (ICI response)	validated; near integration	[59,73,74]
PD-L1	Lung; GU; others	Cytoplasmic/membranous	TPS; CPS; IC score	Cell-type classification; weakly supervised DL	Predictive (ICI response)	validated; assay-dependent integration	[37,39,41,42,63]
Ki-67	Breast; NETs; GU	Nuclear	% positive nuclei	DL nuclear segmentation; automated index	Prognostic	validated; limited exploratory integration	[51,52,60,61,62]

**Table 3:** ML Applications to Emerging Prognostic and Predictive GU IHC Biomarkers.

Biomarker	Cancer	Staining Pattern	Current Role	Emerging Role	Translational Status	ML Involvement	References
AR	Prostate	Nuclear	Predictive marker for endocrine response	Resistance profiling; treatment-stratification	Retrospective/exploratory	Nuclear segmentation and positivity quantification	[110,111]
PTEN	Prostate	Cytoplasmic loss	Prognostic for tumour aggressiveness	Potential therapeutic-decision biomarker	Retrospective/exploratory	Binary loss classification suitable for DL workflows	[53]
PSMA	Prostate	Membranous	Diagnostic/Theranostic	Exploratory prognostic, and predictive of response to ADC or radioligand therapy	Early clinical evaluation	Membrane intensity modelling for automated scoring	[48,112]
MMR proteins	Prostate	Nuclear	Validated predictive marker of ICI response in colorectal cancers	Emerging similar role in some prostate cancer patients	Validated (non-GU); exploratory in GU	Predictive MMR status from H&E; nuclear binary detection of loss vs. retention for IHC	[73,74,86,87]
BAP1	Renal (RCC)	Nuclear loss	Prognostic indicator in RCC	Risk stratification and hereditary context under study	Retrospective/exploratory	Reliable, automated classification of loss vs. retention	[113,114]
VISTA	Bladder, RCC	Immune- associated	Immune context assessment	Candidate predictive biomarker for ICI response	Pre-clinical/hypothesis-generating	Immune-cell compartment segmentation for ML	[115,116]
B7-H3 (CD276)	GU tumours	Membranous	Immune evasion marker	ADC development and therapeutic targeting	Pre-clinical/hypothesis-generating	Membrane-based quantification suited to DL models	[117]
CK5/6, CK14	Bladder	Cytoplasmic/ membranous	Basal subtyping	Exploratory prognostic associations in MIBC	Retrospective/exploratory	ML-based subtype recognition demonstrated	[46,64]
CK20, GATA3	Bladder	CK20 cytoplasmic; GATA3 nuclear	Luminal subtyping	Investigated for recurrence/progression risk	Retrospective/exploratory	Stable staining supports training datasets	[80,108]
UPK II	Bladder	Membranous	Diagnostic; lineage confirmation	Exploratory prognostic associations in MIBC	Retrospective/exploratory	Membrane scoring compatible with DL pipelines	[49]
Nectin-4	Bladder	Membranous	Target of enfortumab vedotin	Exploratory prognostic and predictive for ADC	Early clinical evaluation	ML_automated H-scoring	[118,119,120]
Trop-2	Bladder	Membranous	Target of sacituzumab govitecan	Exploratory IHC-based risk stratification for ADC response	Pre-clinical/hypothesis-generating	Target expression quantification for ML prediction models	[91,118]

## Data Availability

No new data were created or analysed in this study. Data sharing is not applicable to this article.

## Supplementary Materials

The following supporting information can be downloaded at: https://www.mdpi.com/article/10.3390/curroncol33010031/s1, Figure S1. Overview of the Two-Stage Thematic Literature Search Strategy for this Narrative Synthesis; Table S1. AI/ML-based IHC Biomarker Studies Identified in “Stage 1” of the Literature Search.

## Abbreviations

The following abbreviations are used in this manuscript:

AI	Artificial Intelligence
AR	Androgen Receptor
ASCO	American Society of Clinical Oncology
AUC	Area Under the Curve
BAP1	BRCA1-Associated Protein 1
B7-H3	B7 Homolog 3
CAP	College of American Pathologists
CK	Cytokeratin
CPS	Combined Positive Score
CNN	Convolutional Neural Network
ctDNA	Circulating Tumour DNA
DAB	3,3′-Diaminobenzidine
DFS	Disease-Free Survival
dMMR	Deficient Mismatch Repair
ER/PR	Estrogen Receptor/Progesterone Receptor
FFPE	Formalin-Fixed Paraffin-Embedded
GU	Genitourinary
HER2	Human Epidermal Growth Factor Receptor 2
H&E	Hematoxylin and Eosin
H-score	Histologic/Histochemical Score
ICC	Intraclass Correlation Coefficient
ICI	Immune Checkpoint Inhibitor
IHC	Immunohistochemistry
ISH	In Situ Hybridisation
Ki-67	Nuclear proliferation biomarker
LAG-3	Lymphocyte Activation Gene 3
MIL	Multiple Instance Learning
ML	Machine Learning
MLH1, PMS2, MSH2, MSH6	(Mismatch repair proteins)
MMR	Mismatch Repair
MIBC	Muscle-Invasive Bladder Cancer
MSI	Microsatellite Instability
NAC	Neoadjuvant Chemotherapy
NETs	Neuroendocrine Tumours
NSCLC	Non–Small Cell Lung Cancer
PD-1	Programmed Death Receptor 1
PD-L1	Programmed Death-Ligand 1
PI3K	Phosphatidylinositol 3-Kinase
pMMR	Proficient Mismatch Repair
PSMA	Prostate-Specific Membrane Antigen
PTEN	Phosphatase and Tensin Homolog
RCC	Renal Cell Carcinoma
TIM-3	T-cell immunoglobulin and mucin-domain containing-3
TPS	Tumour Proportion Score
Trop-2	Trophoblast cell-surface antigen 2
UPKII	Uroplakin II
UTUC	Upper Tract Urothelial Carcinoma
VISTA	V-domain Immunoglobulin Suppressor of T-cell Activation
WSI	Whole-Slide Imaging
