# Supplementary material for: Machine Learning in Biomarker-Driven Precision Oncology: Automated Immunohistochemistry Scoring and Emerging Directions in Genitourinary Cancers
Source: Curr Oncol. 2026 Jan 6;33(1):31. doi: 10.3390/curroncol33010031 (PMC12840502; doi:10.3390/curroncol33010031)
Supplement: Supplementary file 1 [file curroncol-33-00031-s001.zip › curroncol-4072404-supplementary.pdf]

Supplementary Materials.

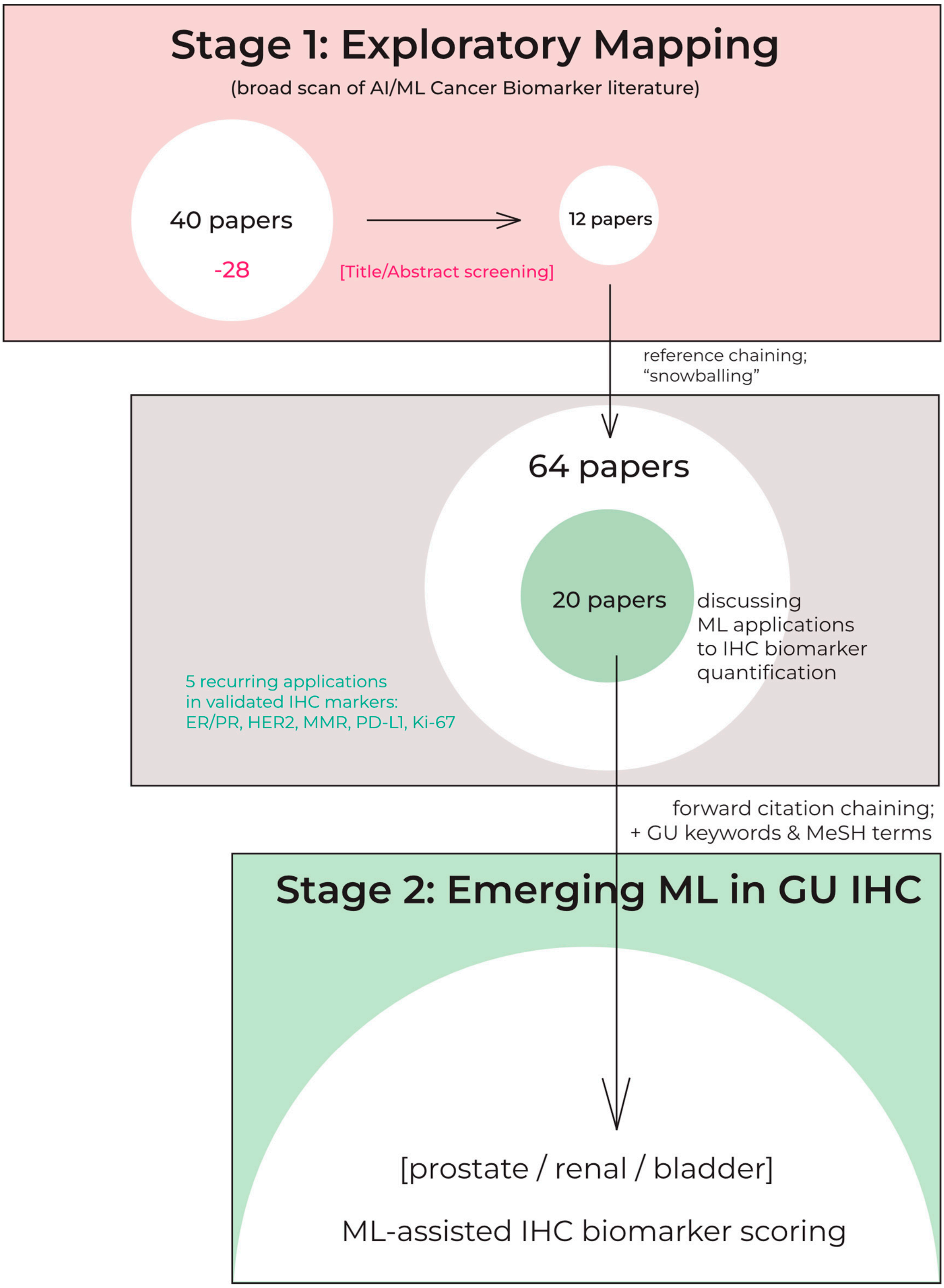

Figure S1. Overview of the Two-Stage Thematic Literature Search Strategy for this Narrative Synthesis

Table S1. AI/ML-based IHC Biomarker Studies Identified in “Stage 1” of the Literature Search

Author/s (Year)	ER/PR	HER2	PD-L1	MMR	Ki-67	Other	Clinical Role
Mulrane et al. (2008) [29]					✓		Prognostic
Polley et al. (2013) [52]					✓		Prognostic
Howat et al. (2014) [31]	✓	✓				EGFR, CK5/6	Diagnostic
Phillips et al. (2015) [37]			✓				Diagnostic / Predictive
Stålhammar et al. (2016) [27]	✓	✓					Diagnostic / Prognostic
Vandenberghe et al. (2017) [30]		✓					Diagnostic
Meyerholz et al. (2018) [50]					✓		Diagnostic / Prognostic
Lakshmi et al. (2019) [105]					✓		Prognostic
Widmaier et al. (2020) [41]			✓				Diagnostic / Predictive
Echle et al. (2020) [82]				✓			Diagnostic / Predictive
Naso et al. (2021) [38]			✓			CD8	Prognostic / Predictive
Baxi et al. (2022) [39]			✓				Prognostic / Predictive
Cheng et al. (2022) [42]			✓				Diagnostic / Predictive
Ivanova et al. (2024) [34]	✓	✓					Prognostic
Wen et al. (2024) [54]					✓		Diagnostic / Prognostic
Nowak et al. (2024) [81]				✓			Prognostic / Predictive
Li et al. (2025) [83]				✓			Predictive
Abukhiran et al. (2025) [104]					✓		Prognostic
Jiao et al. (2025) [95]			✓				Diagnostic / Predictive
Cavalcanti et al. (2025) [103]				✓	✓		Diagnostic / Prognostic / Predictive
